# Supplementary material for: Complex early childhood experiences: Characteristics of Northern Territory children across health, education and child protection data
Source: PLoS One. 2023 Jan 19;18(1):e0280648. doi: 10.1371/journal.pone.0280648 (PMC9851518; doi:10.1371/journal.pone.0280648)
Supplement: S1 Appendix — (DOCX) [file pone.0280648.s001.docx]

**Appendix item 1:** Rationale for inclusion of CPS variables into models

Any contact with child protection services is associated with negative outcomes in a range of areas, as previously discussed. Specifically, the volume of abuse and the chronicity of abuse (number of developmental stages where any notification made for a specific child) affect psychological and educational outcomes [14, 18, 20]. Therefore, in our data-driven analysis, the number of notifications is included to represent volume of abuse, and the notifications are separated by age group to allow chronicity to influence the clusters. Furthermore, in previous work using similar datasets, it has been established that both those notifications which are investigated and substantiated and notifications that are either not investigated or investigated and not substantiated are also important markers of risk, including for later childhood self-harm and juvenile offending compared with those children with no notifications [58, 134]. Thus, both notifications and substantiations are included. Finally, given reporter types include police, CPS, school and healthcare workers, and this project is aiming to understand crossover between these services, including reporter type was considered critical.
