# Supplementary material for: Complex early childhood experiences: Characteristics of Northern Territory children across health, education and child protection data
Source: PLoS One. 2023 Jan 19;18(1):e0280648. doi: 10.1371/journal.pone.0280648 (PMC9851518; doi:10.1371/journal.pone.0280648)
Supplement: S2 Appendix — (DOCX) [file pone.0280648.s002.docx]

**Appendix item 2:** Details on description of K-means

K-means is a simple and efficient algorithm, popular in health research [89]. It is a distance-based algorithm, which follows the below steps:

1. Number of clusters, k. We pre-select the number of clusters either based on content knowledge or cluster number selection techniques, discussed below
2. Initiation step: several cluster centres, *u*, are initially chosen
   1. We used the scikit-learn automatic k-means++ initialisation method for choosing the initial u, which tends to choose points distant from each other, which leads to better results than randomly choosing initial centres [135].
3. The Euclidean distance between each datapoint and each initial cluster centre is calculated, as per below, with N datapoints, x, and K cluster centres, u
4. Assignment/Expectation step: each datapoint is assigned to the cluster it is nearest to (Euclidean distance), to minimise J. The variable r in the equation above represents a binary indicator variable, so that if a datapoint x is assigned to cluster k, $r_{\begin{aligned} nk \\ \end{aligned}}=1,$ or else $r_{\begin{aligned} nk \\ \end{aligned}}= 0$
5. Update/Maximisation step: cluster centres, $u_{k}$ are updated to be the mean of all points that were assigned to that cluster, k (i.e. all points where $r_{\begin{aligned} nk \\ \end{aligned}}=1)$
6. The algorithm continues to move between steps 2 and 3 until convergence is reached, meaning that the assignment of points to a cluster no longer changes
7. K-means is not guaranteed to reach a global minimum, so therefore steps 2-6 are repeated using several different initial cluster centres (in this paper, the k-means was repeated 10 times). The result with the best output in terms of inertia – meaning the lowest sums of squared distances to the cluster centre (*J)* is chosen [135]

The ‘ideal’ number of clusters will result in the most compact and separated clusters. We used the silhouette coefficient to assess this, which follows the below steps:

1. For datapoint *x* in cluster *k*, the mean distance between *x* and all other datapoints in the same cluster *k* is calculated and defined as *a*
2. For datapoint *x* in cluster *k*, the mean dissimilarity score between *x* and another cluster *j* is defined as the average distance between *x* and each datapoint in cluster *j,* defined as *c*
3. The minimum mean dissimilarity score is defined as *b*, meaning that *b* represents the smallest *c* – i.e. the mean dissimilarity score between point *x* and the *nearest neighbouring* cluster
4. The silhouette score for each datapoint is defined as below:

$$Silhouette score=\frac{b-a}{max(a,b)}$$

5. The mean silhouette score for the entire dataset is calculated, for the purposes of cluster number (k) selection. The point at which increasing the number of clusters (clusters from K=2-11 were tested) resulted in diminishing returns in improving the score was chosen from visual inspection of a plot of cluster size vs. silhouette score
