# Supplementary material for: Complex early childhood experiences: Characteristics of Northern Territory children across health, education and child protection data
Source: PLoS One. 2023 Jan 19;18(1):e0280648. doi: 10.1371/journal.pone.0280648 (PMC9851518; doi:10.1371/journal.pone.0280648)
Supplement: S3 Appendix — (DOCX) [file pone.0280648.s003.docx]

**Appendix item 3:** Detail on dimensionality reduction methods

K-means cannot deal with the 'curse of dimensionality', meaning its performance can suffer in high-dimensionality datasets. With increasing dimensions, each observation in the dataset appears similarly distant to all others – rather than simply having to calculate distances across two or three dimensions, distance must be calculated across every dimension – the more dimensions involved, the less significant that any individual distance becomes, and the greater chance that a difference apparent in some dimensions becomes nullified by similarity in another. This can be seen by considering the formula for calculating Euclidean distance between two dimensional vectors with cartesian coordinates *p = (p_1_, p_2_,…p_n_)* and *q = (q_1_, q_2_,…q_n_)*

$$d\left( p,q \right)= \sqrt{\sum_{i=1}^{n} (p_{i}}-q_{i})^{2}$$

Each time a new dimension is added, this adds a non-negative term to the sum, so the distance increases between vectors. This increased distance results in an increasingly sparse feature space. The maximum and minimum distances between points become similar, with ${dist}_{max(A)}$ being the maximum distance between point A and any other point in the dataset and ${dist}_{min(A)}$ being the distance between point A and its closest neighbour, as the number of dimensions, n, increases:

$$\lim_{n\to\infty} {\left( {(dist}_{max(A)}-{dist}_{\min\left( A \right)})/{dist}_{min(A)} \right) \to0}$$

This can result in meaningless clusters for k-means, which relies on Euclidean distance measures.

The impact of this sparsity on supervised machine learning (i.e. classification tasks) is described as ‘Hughes phenomenon’ or the ‘Peaking phenomenon’ – wherein the performance of a model improves, then rapidly deteriorates, as more dimensions are added, if the size of the training dataset remains the same. This is because there are higher combinations of feature values possible, which the classifier cannot learn accurately using the same size training dataset [91].

Although it is still considered a new field, there has therefore been substantial work done on variable subset selection for use in clustering, [93-97]. It is a challenging problem, firstly, because it is unsupervised, so there are no labels to allow for evaluation of variable importance based on classification accuracy (machine learning) or univariate relationship with the outcome (classical statistics). Secondly, because the number of clusters isn’t predetermined, and the cluster number affects variable importance, and vice versa, it is difficult to unpick the entwined issues of cluster number and variable selection [96].
