# Supplementary material for: Complex early childhood experiences: Characteristics of Northern Territory children across health, education and child protection data
Source: PLoS One. 2023 Jan 19;18(1):e0280648. doi: 10.1371/journal.pone.0280648 (PMC9851518; doi:10.1371/journal.pone.0280648)
Supplement: S4 Appendix — (DOCX) [file pone.0280648.s004.docx]

**Appendix item 4:** Detail on the 3 dimensionality reduction methods

*Method 1* is expert selection aka pre-cluster variable filtering. Filtering refers to the use of an expert knowledge pre-processing step, which considers the innate properties of the variables and their presumed relationship to the outcomes of interest to choose a subset [94]. Filtering is specific to the data and research question, but is guided by the principle of diversity – finding a subset of variables that are more diverse and therefore represent the unique aspects of the data without redundancy.

Dimensionality reduction method 1: using filtering, we defined a minimalist, four variable, highly diverse variable set:

- 1. *Year 1 attendance rate*
  2. *Number of hospitalisations*
  3. *Number of neglect notifications*
  4. *Number of notifications for physical, emotional, or sexual abuse*

Notifications rather than substantiations were used. Previous research using NT data has indicated that even unsubstantiated notifications in early childhood carry risk [58]. Children with substantiations in early and middle childhood have a nine times higher risk of self-harm in adolescence than children with no CPS contact, and children with *only notifications in* early childhood and substantiations in middle childhood also have six times higher risk of self-harm in adolescence than children with no CPS contact [58]. CPS notifications were split into abuse and neglect as these are separate entities – one is an act of omission and one is an act of commission and can have differing effects on children’s development [136-138].

*Method 2* is pre-clustering variable extraction via PCA. PCA is a method of automatic dimensionality reduction that projects the original data into a smaller number of dimensions [98]. It can be considered an orthogonal linear transformation that transforms the data into a new coordinate system, wherein the first principal component (PC1) contains the maximal variance in the data, by capturing a lot of information that was previously stored in multiple other variables. PCA creates this new set of orthogonal variables called principle components, by following the steps below [98]:

- 1. Data is standardised
  2. A covariance matrix is calculated, which is *d x d* in size (d being number of dimensions in the dataset)
  3. Eigenvectors are calculated for the covariance matrix. Eigenvectors are vectors that have a direction which does not change when a linear transformation is applied to them. An eigenvalue is the scalar associated with an eigenvector
  4. The eigenvalues are ordered in descending order. The first principal component (PC1) is the eigenvector with the highest eigenvalue. The second principle component is an orthogonal line to PC1 in the two dimensional space [139]. The third principal component is an orthogonal line to PC1 in the third dimensional space, etc.

PCA was carried out using the scikit-learn package, set to reduce the full 43 variable data to 4 dimensions [90]. Whilst PCA has previously been used for dimensionality reduction pre-clustering [140], some suggest that using the principal components of a dataset does not always retain the variables with the most information about cluster membership [93]

*Method 3* is post-clustering variable extraction via decision trees. A post-clustering variable importance analysis was performed to define a third variable subset, following the steps below:

- 1. Cluster using k-means on variable subset (1) and on variable subset (2), , creating two sets of clusters. Assign two cluster memberships to each datapoint
  2. The extra Tree Classifier in scikit-learn (a popular, tree-based, supervised machine learning classifier [90]) was used to predict both sets of cluster labels, using all the original 43 variables as possible predictors
  3. The inbuilt variable importance metric was used to rank the variables based on their contribution to predicting both sets of cluster membership – for details on this variable importance metric, see [99]
  4. The top 10 ranked variables predicting each set of cluster labels were combined to form one variable set of 17 variables (10 + 10 – 3 repeated variables = 17)
  5. The set of 17 variables was reduced with PCA to four dimensions

Dimensionality reduction method 3, final variables:

1. *Year 1 attendance rate*
2. *Number of hospitalisations*
3. *Number of neglect notifications*
4. *Number of neglect substantiations*
5. *Number of hospitalisations with gastroenteritis*
6. *Number of emotional abuse notifications*
7. *Number of emotional abuse substantiations*
8. *Number of overall abuse notifications*
9. *Number of hospitalisations with nutritional deficiency*
10. *Number of substantiations with Domestic Violence as substantiation descriptor*
11. *Number of hospitalisations with skin infection diagnosis*
12. *Number of hospitalisations with infancy related conditions*
13. *Overall number of notifications*
14. *Number of notifications made by police*
15. *Number of notifications made by health professionals*
16. *Number of notifications made from age 4-5 years*
17. *Number of notifications made from age 4-5 years*
