# Supplementary material for: Complex early childhood experiences: Characteristics of Northern Territory children across health, education and child protection data
Source: PLoS One. 2023 Jan 19;18(1):e0280648. doi: 10.1371/journal.pone.0280648 (PMC9851518; doi:10.1371/journal.pone.0280648)
Supplement: S5 Appendix — (DOCX) [file pone.0280648.s005.docx]

**Appendix Item 5:** full model results

| **Model 1:** Expert Selection | | | | | |  | |  |
| --- | --- | --- | --- | --- | --- | --- | --- | --- |
| Feature (median per cluster) | **Non-attenders** | **Thriving** | | **Neglect** | | **Abuse** | | **Ill** |
| Cluster size (%) | 1639 (0.20) | 5361 (0.65) | | 343 (0.04) | | 594 (0.07) | | 330 (0.04) |
| Year 1 attendance rate | 46.07 | 91.45 | | 74.09 | | 84.1 | | 60.42 |
| Number of* hospitalisations by age 5 | 2 | 0 | | 3 | | 2 | | 9 |
| Number of neglect notifications | 0 | 0 | | 4 | | 1 | | 1 |
| Number of neglect substantiations | 0 | 0 | | 1 | | 0 | | 0 |
| Number of hospitalisations with gastroenteritis | 0 | 0 | | 0 | | 0 | | 2 |
| Number of emotional abuse notifications | 0 | 0 | | 0 | | 1 | | 0 |
| Number of emotional abuse substantiations | 0 | 0 | | 0 | | 0 | | 0 |
| Number of abuse notifications | 0 | 0 | | 1 | | 3 | | 0 |
| Number of hospital admissions with nutritional deficiency | 0 | 0 | | 0 | | 0 | | 1 |
| Number of substantiations with Domestic Violence as substantiation descriptor | 0 | 0 | | 0 | | 0 | | 0 |
| Number of hospitalisations with skin infections | 0 | 0 | | 0 | | 0 | | 1 |
| Number of hospitalisations with infancy related conditions | 0 | 0 | | 0 | | 0 | | 1 |
| Number of notifications (any type) to CPS | 0 | 0 | | 5 | | 4 | | 1 |
| Number of notifications made by police | 0 | 0 | | 1 | | 2 | | 0 |
| Number of notifications made by health professionals | 0 | 0 | | 1 | | 0 | | 0 |
| Number of substantiations with Alcohol and Other Drugs as substantiation descriptor | 0 | 0 | | 0 | | 0 | | 0 |
| Number of notifications made from age 4-5 years | 0 | 0 | | 1 | | 1 | | 0 |
| Remote birth hospital (proportion) | 1 | 0 | | 0 | | 0 | | 1 |
| Less than 7 antenatal care visits (proportion) | 0.37 | 0.21 | | 0.51 | | 0.41 | | 0.46 |
| Mother<18 years old (proportion) | 0.14 | 0.04 | | 0.08 | | 0.08 | | 0.13 |
| Born premature (proportion) | 0.13 | 0.08 | | 0.16 | | 0.12 | | 0.26 |
| Born with low both weight (proportion) | 0.12 | 0.06 | | 0.19 | | 0.14 | | 0.27 |
| Maternal alcohol use in pregnancy (proportion) | 0.1 | 0.07 | | 0.33 | | 0.18 | | 0.19 |
| Maternal smoking in pregnancy (proportion) | 0.53 | 0.27 | | 0.66 | | 0.57 | | 0.44 |
| **Model 2:** PCA | | | | | | | |  |
| Cluster name | **Mixed, low vulnerability** | | **Thriving** | | **Mixed, high vulnerability** | | **Ill** |  |
| Cluster size (%) | 1133 (13.7) | 6480 (78.8) | | 345 (4.1) | | 309 (3.7) | |  |
| Feature (median per cluster) |  |  | |  | |  | |  |
| Year 1 attendance rate | 74.19 | 88.91 | | 79.04 | | 55.16 | |  |
| Number of hospitalisations by age 5 | 2 | 1 | | 3 | | 9 | |  |
| Number of neglect notifications | 1 | 0 | | 3 | | 1 | |  |
| Number of neglect substantiations | 0 | 0 | | 1 | | 0 | |  |
| Number of hospitalisations with gastroenteritis | 0 | 0 | | 0 | | 3 | |  |
| Number of emotional abuse notifications | 0 | 0 | | 1 | | 0 | |  |
| Number of emotional abuse substantiations | 0 | 0 | | 1 | | 0 | |  |
| Number of abuse notifications | 1 | 0 | | 3 | | 0 | |  |
| Number of hospital admissions with nutritional deficiency | 0 | 0 | | 0 | | 2 | |  |
| Number of substantiations with Domestic Violence as substantiation descriptor | 0 | 0 | | 1 | | 0 | |  |
| Number of hospitalisations with skin infections | 0 | 0 | | 0 | | 1 | |  |
| Number of hospitalisations with infancy related conditions | 0 | 0 | | 0 | | 1 | |  |
| Number of notifications (any type) to CPS | 2 | 0 | | 6 | | 1 | |  |
| Number of notifications made by police | 1 | 0 | | 2 | | 0 | |  |
| Number of notifications made by health professionals | 0 | 0 | | 1 | | 0 | |  |
| Number of substantiations with Alcohol and Other Drugs as substantiation descriptor | 0 | 0 | | 1 | | 0 | |  |
| Number of notifications made from age 4-5 years | 0 | 0 | | 1 | | 0 | |  |
| Remote birth hospital (proportion) | 0 | 0 | | 0 | | 1 | |  |
| Less than 7 antenatal care visits (proportion) | 0.42 | 0.23 | | 0.49 | | 0.48 | |  |
| Mother<18 years old (proportion) | 0.1 | 0.06 | | 0.06 | | 0.16 | |  |
| Born premature (proportion) | 0.14 | 0.09 | | 0.15 | | 0.22 | |  |
| Born with low both weight (proportion) | 0.15 | 0.07 | | 0.2 | | 0.25 | |  |
| Maternal alcohol use in pregnancy (proportion) | 0.18 | 0.07 | | 0.32 | | 0.2 | |  |
| Maternal smoking in pregnancy (proportion) | 0.56 | 0.31 | | 0.66 | | 0.48 | |  |
| **Model 3:** Post clustering variable extraction | |  | |  | |  | |  |
| Cluster name | **Non-attenders** | **Abuse** | | **Thriving** | | **Neglect** | | **Ill** |
| Cluster size (%) | 909 (0.12) | 221 (0.03) | | 6346 (0.77) | | 191 (0.02) | | 600 (0.07) |
| Feature (median per cluster) |  |  | |  | |  | |  |
| Year 1 attendance rate | 77.08 | 82.15 | | 89.3 | | 73.82 | | 54.36 |
| Number of hospitalisations by age 5 | 2 | 2 | | 1 | | 4 | | 6 |
| Number of neglect notifications | 1 | 2 | | 0 | | 4 | | 1 |
| Number of neglect substantiations | 0 | 0 | | 0 | | 2 | | 0 |
| Number of hospitalisations with gastroenteritis | 0 | 0 | | 0 | | 1 | | 2 |
| Number of emotional abuse notifications | 1 | 2 | | 0 | | 0 | | 0 |
| Number of emotional abuse substantiations | 0 | 1 | | 0 | | 0 | | 0 |
| Number of abuse notifications | 1 | 4 | | 0 | | 1 | | 0 |
| Number of hospital admissions with nutritional deficiency | 0 | 0 | | 0 | | 0 | | 1 |
| Number of substantiations with Domestic Violence as substantiation descriptor | 0 | 2 | | 0 | | 0 | | 0 |
| Number of hospitalisations with skin infections | 0 | 0 | | 0 | | 0 | | 1 |
| Number of hospitalisations with infancy related conditions | 0 | 0 | | 0 | | 0 | | 1 |
| Number of notifications (any type) to CPS | 2 | 5 | | 0 | | 5 | | 1 |
| Number of notifications made by police | 1 | 3 | | 0 | | 1 | | 0 |
| Number of notifications made by health professionals | 0 | 0 | | 0 | | 2 | | 0 |
| Number of substantiations with Alcohol and Other Drugs as substantiation descriptor | 0 | 0 | | 0 | | 1 | | 0 |
| Number of notifications made from age 4-5 years | 1 | 1 | | 0 | | 1 | | 0 |
| Remote birth hospital (proportion) | 0 | 0 | | 0 | | 0 | | 1 |
| Less than 7 antenatal care visits (proportion) | 0.4 | 0.45 | | 0.23 | | 0.52 | | 0.48 |
| Mother<18 years old (proportion) | 0.1 | 0.06 | | 0.06 | | 0.06 | | 0.15 |
| Born premature (proportion) | 0.12 | 0.14 | | 0.08 | | 0.14 | | 0.23 |
| Born with low both weight (proportion) | 0.12 | 0.18 | | 0.07 | | 0.2 | | 0.26 |
| Maternal alcohol use in pregnancy (proportion) | 0.17 | 0.21 | | 0.07 | | 0.41 | | 0.18 |
| Maternal smoking in pregnancy (proportion) | 0.54 | 0.64 | | 0.3 | | 0.68 | | 0.51 |
|  |  |  | |  | |  | |  |

*’Number of’ refers to total number from age 0-5, unless otherwise specified
